# Editorial: Frontiers in Chemistry: rising stars 2022

**DOI:** 10.3389/fchem.2023.1334117

**Published:** 2023-11-20

**Authors:** Carlos D. S. Brites, Daniele Contini, Xiao-Peng He, Tianqing Liu, Basem Moosa, Marianna Pannico, Mohamed Nageeb Rashed, Yunping Qiu, Clemens Zwergel

**Affiliations:** ^1^ Phantom-G, Department of Physics, CICECO—Aveiro Institute of Materials, University of Aveiro, Aveiro, Portugal; ^2^ Institute of Atmospheric Sciences and Climate, ISAC-CNR, Lecce, Italy; ^3^ Key Laboratory for Advanced Materials and Joint International Research Laboratory of Precision Chemistry and Molecular Engineering, Feringa Nobel Prize Scientist Joint Research Center, Frontiers Center for Materiobiology and Dynamic Chemistry, School of Chemistry and Molecular Engineering, East China University of Science and Technology, Shanghai, China; ^4^ NICM Health Research Institute, Western Sydney University, Westmead, NSW, Australia; ^5^ Smart Hybrid Materials (SHMs) Laboratory, Advanced Membranes and Porous Materials Center, Division of Physical Sciences and Engineering, King Abdullah University of Science and Technology (KAUST), Thuwal, Saudi Arabia; ^6^ National Research Council of Italy, Institute for Polymers, Composites and Biomaterials, Pozzuoli, Italy; ^7^ Faculty of Science, Aswan University, Aswan, Egypt; ^8^ Department of Medicine, Albert Einstein College of Medicine, Bronx, NY, United States; ^9^ Department of Drug Chemistry and Technologies, Sapienza University of Rome, Rome, Italy

**Keywords:** rising stars, chemical sciences, analytical chemistry, medicinal chemistry, organic chemistry

As guest editors of the 2022 Edition of our *Frontiers in Chemistry* “Rising Stars” article collection, we are delighted to showcase a collection of high-quality work of internationally recognized researchers in the early stages of their independent careers, spanning from analytical chemistry over medicinal chemistry to organic chemistry. In the present Research Topic, not only in original articles were submitted, but also numerous reviews are highlighting the latest progress in the field of chemistry in its broadest sense.

The review of Qiu et al. reported the different studies on the high-throughput mass spectrometry metabolomics analytical platforms and software for identifying metabolites and delineating their contribution. These include metabolic profiling of candidate metabolites with different techniques, which include LC/MS, open-source software Skyline tool mass spectrometry (MS), NOREVA that facilitate optimizing the multiclass metabolomic data processing, and the online MetaboAnalyst platform, which is a tool for high-resolution mass spectrometry spectra processing.

The rhizome of Polygonatum odoratum (PO) can be used to treat diabetes and cough. The chemical identification of its two major commercial specifications for this traditional Chinese medicine: Xiangyuzhu (XPO) and Guanyuzhu (GPO), is to be established. In their original research article, Nan et al. developed a comprehensive qualitative and quantitative procedure to differentiate these two specifications. The methods combined ultra-high-performance liquid chromatography-quadruple time-of-flight mass spectrometry (UHPLC-Q-TOF/MS) and UPLC-charged aerosol detection (UHPLC-CAD). The authors discovered that the concentration ratio of timosaponin H1/polygodoraside G is a potential biomarker to differentiate these two specifications.

In their work, Pandit et al. focused on the impact of a single point mutation, particularly in the dynamic C-terminal region of the α-synuclein or alpha-synuclein (aS) protein whose aggregates are linked to Parkinson’s disease. Their study demonstrated that mutating a crucial residue in the C-terminal region of aS with a residue promoting helix formation reduced the protein’s aggregation and harmful effects on neuronal cells.

Scarpellini et al. explored the chemical space around their previously described compound GSK2656157, a known highly potent inhibitor of Protein Kinase (PKR) like ER Kinase (PERK). The structurally related Receptor-Interacting serine/threonine-Protein Kinase 1 (RIPK1) has been identified as a major cause of inflammation and, as a result, of inflammatory pathologies. Their structural optimization of the GSK2656157 scaffold resulted in a new class of more selective RIPK1 inhibitors. Based on the structure-activity relationship reported in the literature, they hypothesized that substituent insertion on the para position of the pyridinyl ring would reduce the interactions with PERK. Subsequent synthesis and evaluation of various para-substituted analogs proved their hypothesis and revealed UAMC3861 as the best compound with a potent RIPK1 inhibitory activity and good selectivity over PERK. Furthermore, UAMC3861 possesses the ability to inhibit RIPK1-dependent apoptosis and necroptosis in an *in vitro* inflammation model.

In their work, Chen et al. discussed the incorporation of silicon (Si) elements into carbon-based compounds, resulting in unique biological and physical-chemical properties. This review provides a summary of recent advancements in silacycle synthesis, including the use of transition metal-catalytic and photocatalytic strategies with various silicon-containing starting materials. It also highlights the mechanistic aspects and features of these developed reaction methodologies.

Precision medicine requires efficient and informative diagnostic tools to deeply understand diseases at molecular levels. Nanoplasmonic sensing is one of the techniques that showed great potential in providing high sensitivity and real-time information to characterize patients for personalized treatment. In this review article, Xiao et al. described the advantages and disadvantages of different nanoplasmonic and analysis-based sensing, the integration of multiple technologies, and the application and future development of nanoplasmonic biosensors in precision medicine. The great sensitivities of nanoplasmonic biosensors allow us to observe and quantify single molecular binding events. With further development, the next generations of nanoplasmonic technology will enrich the information and knowledge for precision medicine.

Nitroguaiacols, organic compounds released into the atmosphere from biomass burning, photolyze under sunlight to form various products, including chromophores. Delić et al. investigated the photolysis kinetics and product formation of two nitroguaiacols, 4-nitroguaiacol (4NG) and 5-nitroguaiacol (5NG), under artificial sunlight. Delić et al. found that 5NG photolysis is slower than 4NG photolysis and produces more chromophores, suggesting that 5NG has a stronger potential for secondary BrC formation. Their findings suggest that 5NG may be a more important precursor to secondary BrC (potent ozone-depleting substances) formation than 4NG. This study provides new insights into the photochemistry of nitroguaiacols and their potential role in forming secondary BrCs in the atmosphere.

Due to its minimal invasiveness and high tissue penetration depth, sonodynamic therapy (SDT) has recently emerged as a promising phototherapeutic modality for cancer. In their review, Hwang et al. summarized the recent progress in the design and synthesis of mitochondria-targeted organic SDT agents capable of being activated by ultrasound to generate cytotoxic reactive oxygen species in deep tumor regions. They also discussed the current challenges and future directions of developing organic SDT agents suitable for clinical settings.

Baltrukevich and Bartos performed Molecular Dynamics (MD) simulations, investigating the interactions and dynamics of, in their case, RNA and various proteins at the atomic level. To date, there have only been a few studies conducted on the simulation of complexes of RNA and proteins in MD simulations. In their paper, they examined the differences in force fields when simulating complexes of RNA and proteins. Specifically, they tested and compared three Non-Polarizable Force Fields (NPFs). All tested models can be used to simulate RNA and protein complexes; however, the NPF needs to be adapted to the research question.

Wurnig et al. designed, synthesized, and evaluated a geldanamycin-based degrader of heat shock protein 90 (HSP90) using the PROTAC strategy to fight cancer. PROTACs degrade the target protein rather than inhibiting it. This strategy is highly relevant in HSP90-related biology as, despite the initial clinical promise, resistance and dose-limiting toxicities have prevented classical HSP90 inhibitors from being used as anticancer drugs. Their best peptide degrader (compound **3a**) effectively reduced HSP90α and HSP90β levels, providing a promising novel approach to target HSP90-driven diseases such as specific forms of cancer.

The work of Meissner et al. investigates the application of temperature-swing solvent extraction (TSSE) as a cost-effective and versatile technology for effectively removing selenium oxyanions and traces of mercury in the presence of high contents of chloride and sulfate. The study shows that TSSE might provide a technological solution with a high deionization potential for the industry in complying with regulations for discharge streams from coal-fired power facilities.

Al-Bukahri et al. reported a Polycondensation of tetrakis (4-aminophenyl) methane with pyrrole and phenazine, yielding newly designed 3D porous polymers. The novel polymers exhibited permanent porosity, with BET surface areas of 575 and 389 m^2^/g, respectively. Solid-state NMR spectroscopy, Fourier-transform infrared (FT-IR) spectroscopy, and thermogravimetric analysis were used to investigate the structure (TGA). The adsorption capacities of the synthesized polymers for CO_2_ and H_2_ were determined to be 1.85 and 2.10 mmol/g, respectively. The significance of the synthesized polymers lies in their CO_2_/N_2_ selectivity, which is 43 and 51, respectively. At 1 bar and 77 K, the polymers demonstrated promising hydrogen storage capacities of 66 cm^3^/g (0.6 wt%) and 87 cm^3^/g (0.8 wt%), respectively.

“*Frontiers in Chemistry: Rising Stars*” is a prestigious compilation of research articles and reviews that showcases the work of upcoming researchers in the field of chemistry. Within this Research Topic, we have seen numerous articles from young promising scientists from all over the world exhibiting the numerous facets of chemistry. The innovative research presented herein has been recognized for its potential to drive advancements and shape the future of the discipline. The publication provides a platform for these emerging scientists to share their findings and insights, which not only contribute to the current body of knowledge in chemistry but also pave the way for innovative discoveries. It acknowledges their significant achievements and celebrates their potential to become future leaders in the field.

